# Evaluating the quantity and spatial density of macrophage-like cells in patients with retinal vascular disease and healthy subjects via non-invasive retinal imaging

**DOI:** 10.1186/s40942-025-00729-7

**Published:** 2025-10-09

**Authors:** Farhad Ghaseminejad, Thomas J. van Rijssen, Parsa Khatami, Pedro L. Rissoli, Ricky Chen, Yudan Chen, Brendan Tao, Myeong Jin Ju, Faisal Beg, Eduardo V. Navajas

**Affiliations:** 1 Department of Ophthalmology & Visual Sciences, University of British Columbia, Vancouver, BC Canada; 2 Department of Ophthalmology, Leiden University Medical Center, Leiden, Netherlands; 3 School of Engineering, Simon Fraser University, Vancouver, BC Canada; 4 Department of Ophthalmology & Vision Sciences, University of Toronto, Toronto, ON Canada; 5 Eye Care Centre, Vancouver General Hospital, British Columbia, 2550 Willow Street, Vancouver, V5Z 3N9 Canada

## Abstract

**Objective:**

Here we investigate the spatial density and distribution of macrophage-like cells (MLCs) in ischemic retinal diseases, including branch retinal vein occlusion (BRVO), central retinal vein occlusion (CRVO), and proliferative diabetic retinopathy (PDR), compared to healthy controls.

**Methods:**

In this pilot investigation with prospective cross-sectional design, OCT angiography (OCTA) images were obtained from 20 eyes across four groups (BRVO, CRVO, PDR, controls). Using a standardized semi-automated image processing protocol, MLCs were identified and quantified over a 6 × 6 mm area temporal to the macula. Additionally, perfusion densities were measured, and analyses were performed to assess differences in MLC counts and their correlations with perfusion.

**Results:**

Patients with BRVO, CRVO, and PDR exhibited significantly higher MLC counts compared to controls (*p* = 0.002 for all comparisons). Median MLC counts were lowest in controls (132; 95% CI, 63–180), followed by BRVO (382; 95% CI, 290–446), CRVO (688; 95% CI, 507–716), and highest in PDR patients (973; 95% CI, 805–999). A moderate-to-strong negative correlation was found between perfusion density and MLC count, suggesting increased MLC accumulation in areas of reduced perfusion in the superior vascular complex (*p* = 0.04), deep vascular complex (*p* < 0.01), as well as combined vascular complexes (*p* = 0.01).

**Conclusion:**

Here, we demonstrate that MLC density is significantly elevated in BRVO, CRVO, and PDR compared to healthy eyes and is inversely correlated with retinal perfusion. By applying consistent imaging and analysis across a wider peripheral retinal field, these findings highlight MLC quantification as a potential biomarker for disease severity and progression in ischemic retinopathies. Future investigations should explore whether modulating MLC responses could offer new therapeutic strategies to improve outcomes in these conditions.

**Supplementary Information:**

The online version contains supplementary material available at 10.1186/s40942-025-00729-7.

## Introduction

As we better understand the pathophysiology of different retinal vascular diseases, the role of Macrophage-Like Cells (MLCs) in the inflammatory response of the retina is increasingly acknowledged. MLCs refer to a variety of cells including microglia, perivascular macrophages, and vitreal hyalocytes [1]. These cells are predominantly located both posteriorly in the vitreous cortex close to the inner limiting membrane and anteriorly near the ciliary body. To a lesser extent, they can also be found as free cells circulating in the vitreous [2]. The roles of MLCs include but are not limited to synthesis of extracellular matrix, modulation of immune reactions, and regulation of inflammatory responses [3, 4]. These processes contribute to maintaining the transparency of the vitreous as well as the expression of anti-vascular endothelial growth factor (Anti-VEGF) under hypoxic conditions to prevent growth of vessels [5, 6]. Important roles for MLCs have been suggested in various eye diseases such as uveitis, tractional vitreoretinopathies, proliferative diabetic retinopathy, and exudative age-related macular degeneration [7]. It has been postulated that targeting MLCs in the early stages of disease may mitigate the progression to advanced disease complications [8, 9].

Adaptive optics optical coherence tomography (AO-OCT) and en-face optical coherence tomography angiography (OCTA) allow for detailed imaging of the retina and the posterior vitreous interface, including MLCs [10, 11]. Although AO-OCT is a strong visualization tool, its use in the clinical setting remains limited due to system complexities and associated costs [12]. OCTA, however, has shown to be a robust, relatively accessible, and non-invasive diagnostic tool with increasing clinical benefit.

Previous studies have reported an increase in the density of MLCs in patients with diabetic retinopathy, nonarteritic anterior ischemic optic neuropathy, and retinal vein occlusion [13–15]. Higher MLC count has also been found to be associated with a higher number of anti-VEGF injections and lower visual acuity in patients with retinal vein occlusions [15]. However, different studies have utilized differing approaches to identifying and quantifying MLCs in each of the diseased states. In this pilot study, we evaluate the spatial density of MLCs and compare the number of MLCs in patients with branch retinal vein occlusion (BRVO), central retinal vein occlusion (CRVO), and proliferative diabetic retinopathy (PDR) as well as healthy subjects using a consistent imaging and analysis methodology. We focus our attention on a larger field of view in the peripheral retina, allowing for a novel and more holistic view of the role of MLCs in ischemic diseases of the retina. Lastly, we evaluate the spatial density of MLCs around the retinal vasculature and assess the association between MLC count and retinal perfusion density in different disease states – a novel approach that has not previously been reported.

## Methods

### Study participants

This cross-sectional, prospective study included patients with diagnosed BRVO, CRVO and PDR as well as healthy controls. Study’s protocol was reviewed and approved by the institutional review board at the University of British Columbia (REB H15-02914) and abides, in full, to the principles of the Declaration of Helsinki. Patients were recruited during their regular appointment at the Eye Care Centre (Vancouver General Hospital, Vancouver, British Columbia, Canada) between April 2021 to October 2022. Written and verbal informed consents were obtained from all study participants.

Patients’ diagnoses of PDR, CRVO, BRVO, or healthy control were determined by a board-certified vitreoretinal surgeon and the senior author, Dr. Eduardo Navajas, through comprehensive clinical and imaging (fluorescein angiography, OCT, and OCA) evaluation. Participants aged 18 or older were included in the study. The exclusion criteria were as follows: (i) media opacities compromising imaging quality; (ii) active intraocular inflammation; (iii) the presence of other retinal comorbidities (e.g., age-related macular degeneration); (iv) intraocular surgery within three months prior to imaging; and (v) concurrent systemic drug therapy known to cause ocular side effects.

### Image acquisition

OCTA images were obtained using the PLEX™ Elite 9000 (Carl Zeiss Meditec, Dublin, CA), a swept-source OCTA (SS-OCTA) device equipped with a 100-kHz light source, a central wavelength of 1060 nm, and a 100 nm bandwidth. This system achieved an axial resolution of approximately 5.5 μm and a lateral resolution of around 20 μm at the retinal surface. Five 6 × 6 mm images were obtained at two locations surrounding the macular and temporal to the macula, in one eye per patient. Images were re-acquired for cases where image signal strength was poor (< 7/10 as evaluated by the imaging device), or where motion artifacts were noted qualitatively by the technician.

### Image registration and retinal layer segmentation

After the acquisition of OCT data, the volumes from one eye underwent 3D registration and averaging algorithms to enhance the image contrast [16]. The averaging performed improves signal-to-noise ratio and has previously been established to enhance visualization of macrophage-like cells above the ILM [17, 18].In brief, one volume with minimal motion artifacts was selected as a template. To ensure consistency across all participants and to avoid selection bias, the region temporal to the macula was selected as the target area in the peripheral retina, regardless of disease group or the location of retinal involvement. Transformation matrices of the volumes (targets) for 2D registration were estimated based on the en face OCTA image of the template. The matrices were then applied to the OCT volumes of the targets and the axial matching algorithm based on OCT B-scan was applied [19]. Once the 3D registration was completed, the five OCT volumes were averaged to enhance the overall contrast, facilitating a more reliable visualization of MLCs. The averaged volumes were segmented to delineate retinal layers using a pre-trained deep neural network-based model [20].

### Image analysis

MLCs were identified and isolated from the enhanced vitreous imaging OCT en face slab using a semiautomated binarization process with a FIJI macro, as described in a previous study by Ong et al.^13^. This procedure has been reported to have both good intra- and interobserver variability coefficients (0.998 and 0.995, respectively) [13]. The process included noise reduction to remove background irregularities and vessel artifacts, signal enhancement to improve cell identification, and binarization to extract discrete cell shapes [14].

**Fig. 1:**
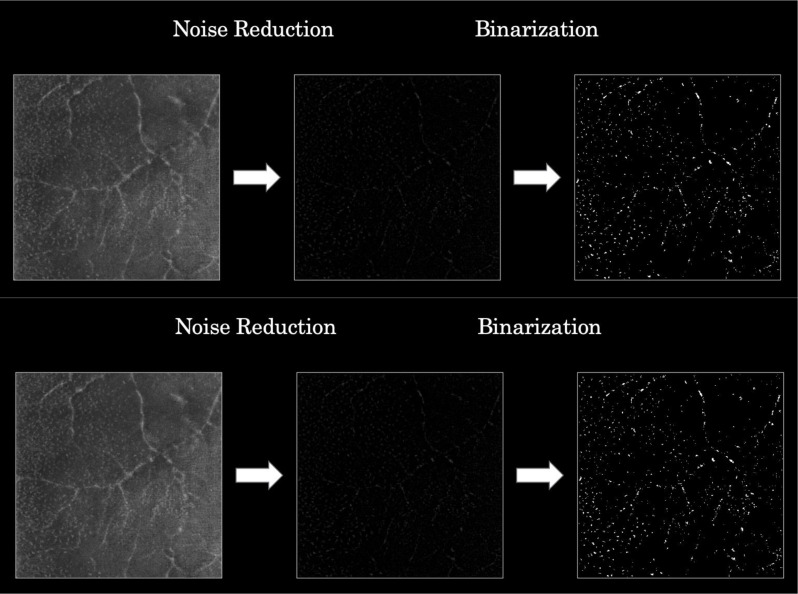
The process of macrophage like cell extraction, noise reduction and binarization in order to reliably visualize and quantify the number of cells in a 6 × 6 mm area around the fovea (top row) and peripheral to the macula (bottom row)

The 25-pixel slab above the inner limiting membrane (ILM) containing MLCs was then processed using ImageJ [17]. The ‘subtract background’ feature was used to reduce image artifact, followed by ‘Max Entropy’ thresholding [13, 21]. The image was then binarized, illustrating MLCs on a black background. The same methodology was applied to images from both the macula and peripheral to the macula (Fig. 1). The ‘Analyze Particle’ feature was utilized to quantify the number of cells in each image [22].

Stack images with MLCs overlaying the blood vessels were created using the ImageJ software. OCTA images of the corresponding 6 × 6 mm area for each patient were made binary (black vessels on white background). OCTA images were resized to match the registered and averaged slab image of MLCs. The Merge Channel function was utilized to combine the images with the blood vessels marked as red and MLCs marked as green.

R-Studio and SPSS Statistics (IBM Corp. version 29.0. Armonk, New York, United States of America) were used for statistical analyses. Non-parametric Kruskal-Wallis and non-parametric Mood’s median tests with stepwise comparisons were used to compare the number of MLCs between the groups. Non-parametric statistics, including Kendall’s tau correlation, were used to assess the relationship between MLC count and perfusion density from individual eyes. For this pilot study, the decision to use Kendall’s tau was based on the expectation of a smaller sample size, which based on current evidence, is better evaluated with a non-parametric test which empirically has the smallest sample size requirement for performance [23]. All analyses were carried out at the levels of the superior vascular complex, the deep vascular complex, and altogether through both complexes.

### Perfusion density analysis

A previously published deep learning technique was employed to quantify retinal vessel perfusion densities [24]. This method assigns probabilities to each pixel, indicating whether it represented a vessel or non-vessel. Following segmentation, probability maps were binarized and perfusion density was defined as the percentage of vessel-containing image area relative to the total image area.

The default segmentation settings were used to delineate the superficial vascular complex (SVC) and deep vascular complex (DVC). The SVC included the radial peripapillary capillary and superficial capillary plexus located between the inner limiting membrane and the posterior boundary of the inner plexiform layer (IPL). The DVC included the intermediate capillary plexus and deep capillary plexus from the posterior boundary of the IPL to the posterior bound of the outer plexiform layer. Associations between MLC counts and perfusion density across vascular complexes were considered exploratory analyses.

## Results

A total of 20 eyes of 20 patients (9 male and 11 female) were included in this study, including 5 eyes in each subgroup (controls, BRVO, CRVO, and PDR) (Table 1; Supplementary Table 1). Median age was 67 years (range 26, 80). The median visual acuity in the control group was 0.00 (range 0.18 to 0.00). In the BRVO group, median visual acuity was 0.34 (range 0.34 to 0.14). In the CRVO group, median visual acuity was 0.14 (range 1.00 to 0.00). Median visual acuity was 0.34 (range 0.34 to 0.14) in the PDR group.

The median number of MLCs in the control group was 130 (range 63–311), while this was 382 (range 290–690) in the BRVO group, 688 (range 507–1140) in the CRVO group, and 969 (range 805–999) in the PDR group. There was a significant difference in the number of MLCs between the control and BRVO group (*p* = 0.002), between the control and CRVO group (*p* = 0.002), and the control and PDR group (*p* = 0.002). There was no significant difference between the BRVO and CRVO groups regarding MLCs count (*p* = 0.058), and between the BRVO and PDR group (*p* = 0.058).

Median MLC counts were lowest in controls (130; 95% CI, 63–180), followed by BRVO (382; 95% CI, 290–446), CRVO (688; 95% CI, 507–716), and highest in PDR patients (973; 95% CI, 805–999). Compared to controls, median MLC counts were higher in all disease groups, with differences of 252 for BRVO (percentile CI 71–560; BCa CI − 21–303), 558 for CRVO (percentile CI 327–1010; BCa CI 196–558), and 839 for PDR (percentile CI 625–910; BCa CI 494–789) (Supplementary Table 2).

**Table 1:** Subject group demographics

		Controls	BRVO	CRVO	PDR
Age median in years (range)		27 (26, 71)	71 (67, 80)	72 (33, 77)	52 (32, 72)
Hyalocyte count median (range)		130 (63, 311)	382 (290, 690)	688 (507, 1140)	969 (805, 999)
Best-corrected visual acuity in Snellen (range); [LogMar]		20/20 (20/30, 20/20); [0.00 (0.18, 0.00)]	20/40- (20/40-, 20/30+); [0.34 (0.34, 0.14)]	20/25- (20/200, 20/20); [0.14 (1.00, 0.00)]	20/40- (20/40-, 20/30+); [0.34 (0.34, 0.14)]
Sex	Male	3	3	1	2
	Female	2	2	4	3

**Fig. 2:**
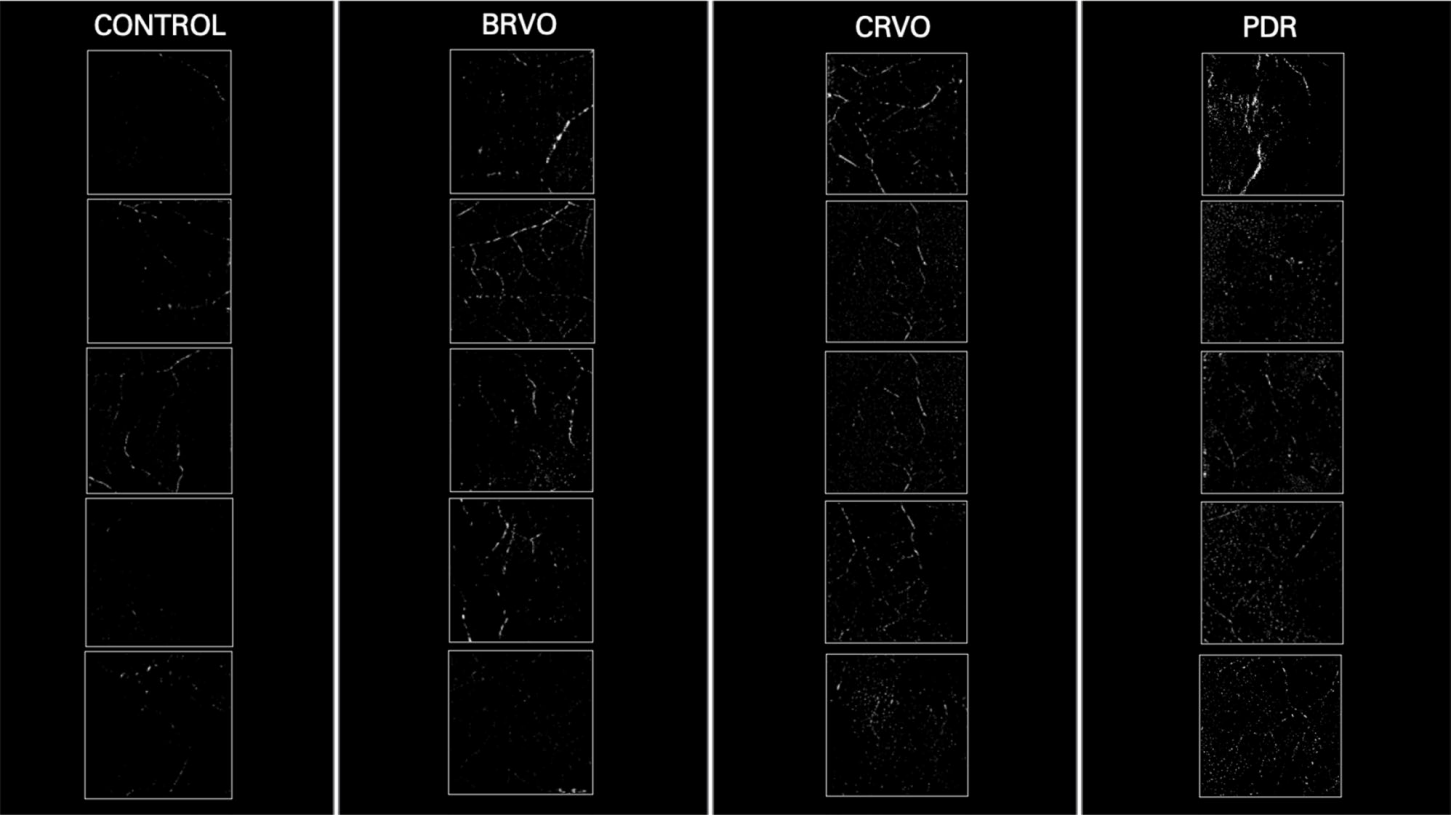
Macrophage-like cells identified in a 6 × 6 mm area in 25 pixel slabs above the inner limiting membrane in peripheral retina in control subjects and patients with branch retinal vein occlusion, central retinal vein occlusion, and proliferative diabetic retinopathy. Each square photo with white border represents one case

**Fig. 3:**
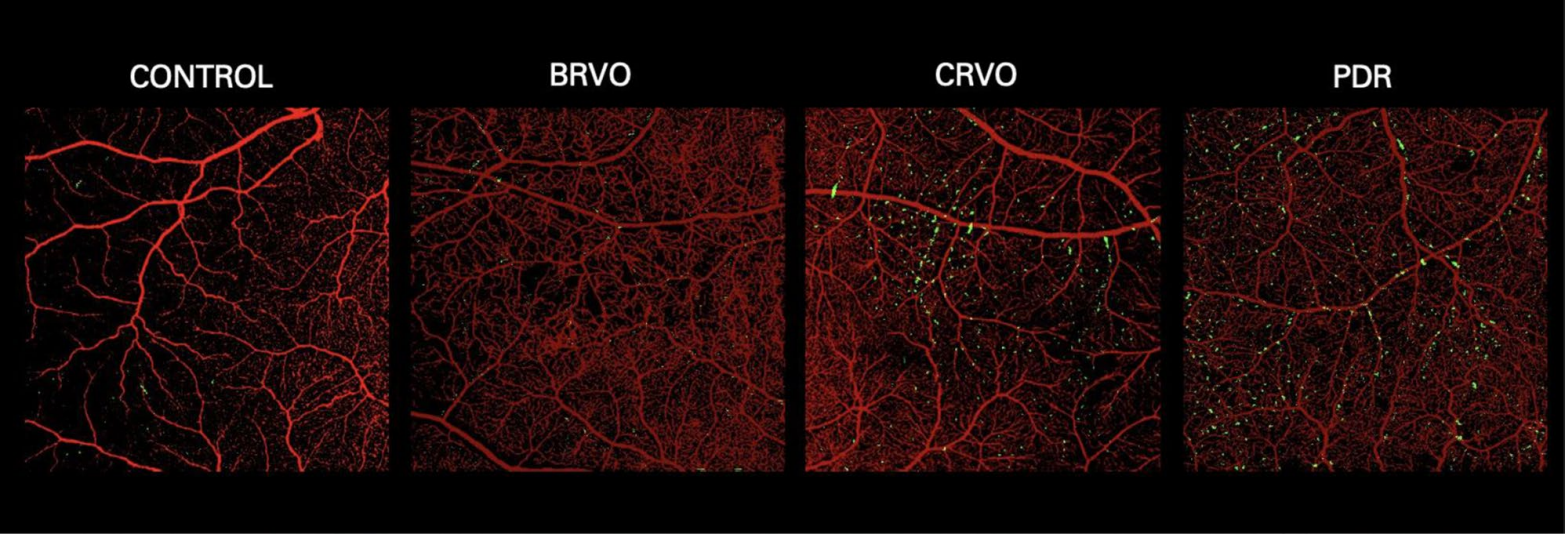
Overlay of macrophage-like cells (MLC) over the vascular complex. MLCs are highlighted in green over the imaged blood vessels shown in red

On qualitative visualization of MLCs, we noted an increasing trend in the number of MLCs imaged in ischemic diseases of the retina, with PDR with the largest number of cells followed by CRVO and BRVO patients (Fig. 2). MLCs were particularly found to accumulate near the retinal vasculature, a pattern that remained consistent amongst all four groups (Fig. 3).

**Fig. 4:**
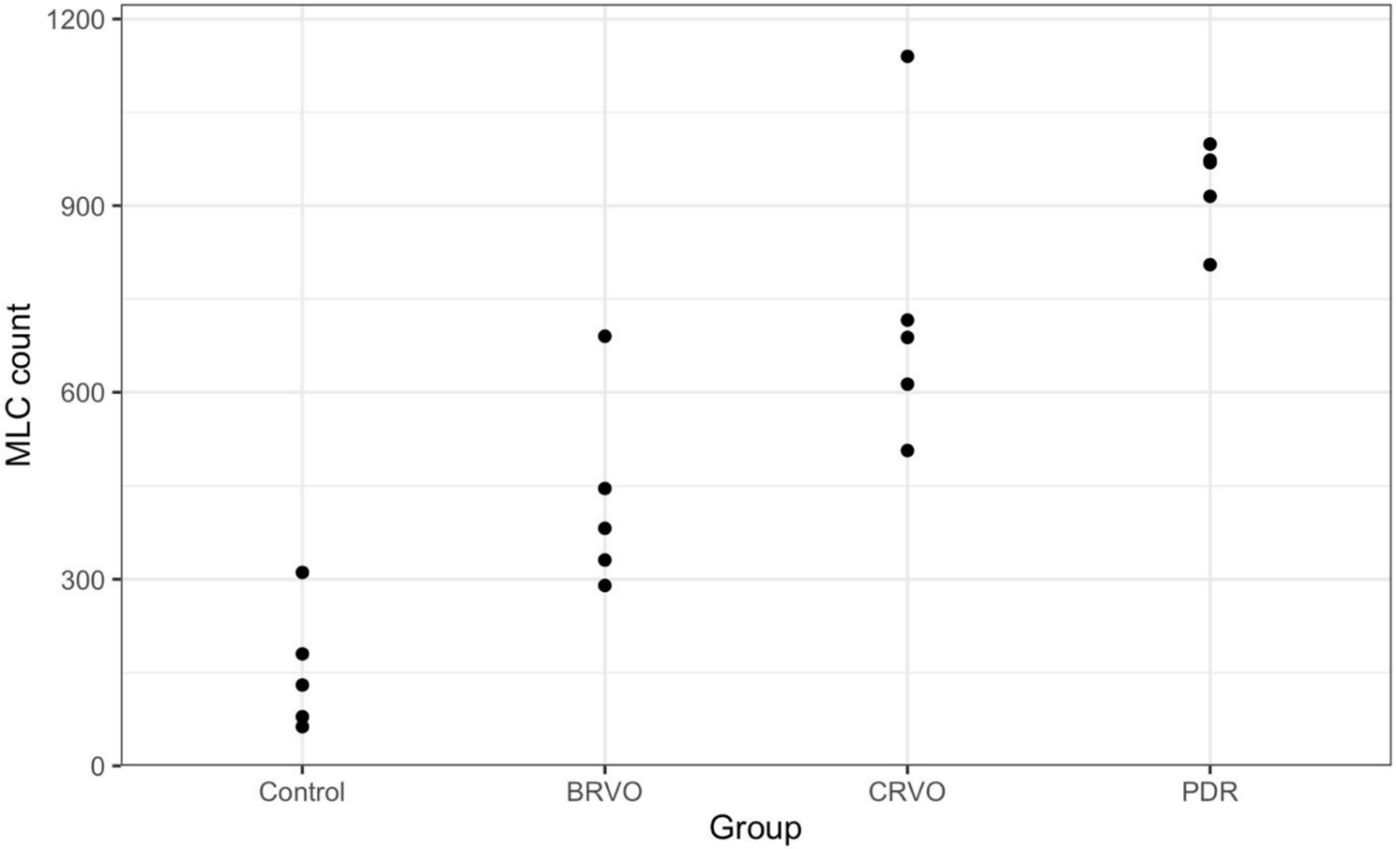
Number of MLCs in each group (*n* = 5 per group). Kruskal-Wallis Test *P* = 0.001*

Following a qualitative comparison, we proceeded to quantify the number of MLCs in each image, allowing for a more objective mode of comparison between groups. Average number of MLCs quantified in a 6 × 6 mm area temporal to the macula are presented in Fig. 4. On average, total MLC count was found to be the highest in the PDR group (*n* = 932), followed by CRVO patients (*n* = 733) and BRVO patients (*n* = 428). MLC count was the lowest in the healthy control group with an average of 153 cells.

Perfusion density was also compared between the groups. The median (interquartile range) perfusion density was 0.50 (0.05) for the BRVO group, 0.43 (0.02) for the CRVO group, 0.42 (0.04) for the PDR group, and 0.52 (0.005) for the control group. Thus, perfusion density was generally lower in the diseased groups compared to control subjects. Considering the association between MLCs and the vascular complex, MLC count was significantly and negatively correlated with perfusion density in the superior vascular complex (*p* = 0.04, tau = -0.49), deep vascular complex (*p* < 0.01, tau = -0.64), as well as combined vascular complexes (*p* = 0.01, tau = -0.6). After applying Bonferroni correction for three comparisons (adjusted α = 0.017), only the associations with the deep and combined vascular complexes remained significant, while the correlation with the superior vascular complex did not (Supplementary Table 3). A scatter plot of MLC versus perfusion density, stratified by retinal layer, is included in the supplement (Supplementary Fig. 1). In a *post hoc* sensitivity analysis, the relationship between perfusion density and MLC count was evaluated using the Spearman’s rho method. MLC count remained significantly and negatively correlated with perfusion density in the deep vascular complex (*p* < 0.01, tau = -0.81) and combined vascular complexes (*p* = 0.01, tau = -0.75). However, by a small margin of 2% compared to the original analysis, MLC was no longer significantly associated with perfusion density in the superior vascular complex (*p* = 0.06, tau = -0.58).

**Fig. 5:**
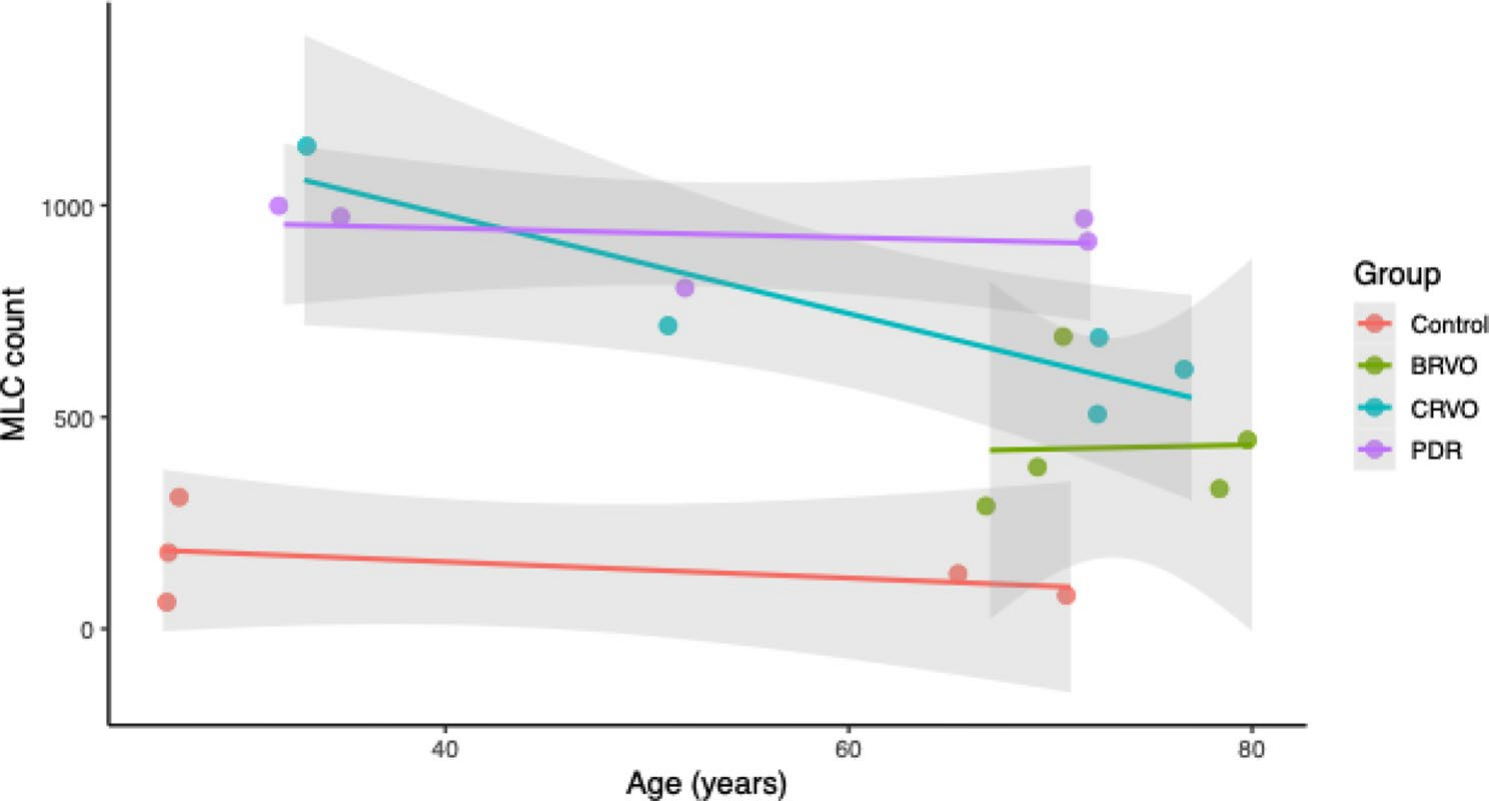
Scatter plots of the relationship between MLC count and age by group. Each point represents an individual eye. Colored regression lines with shaded 95% confidence intervals illustrate trends within each group

MLC count was also examined in relation to age across disease groups. Regression analyses did not reveal a significant positive association between age and MLC count in any group. Instead, trends were flat or negative in controls, BRVO, and CRVO, while PDR showed a relatively stable pattern across age (Fig. 5).

**Fig. 6:**
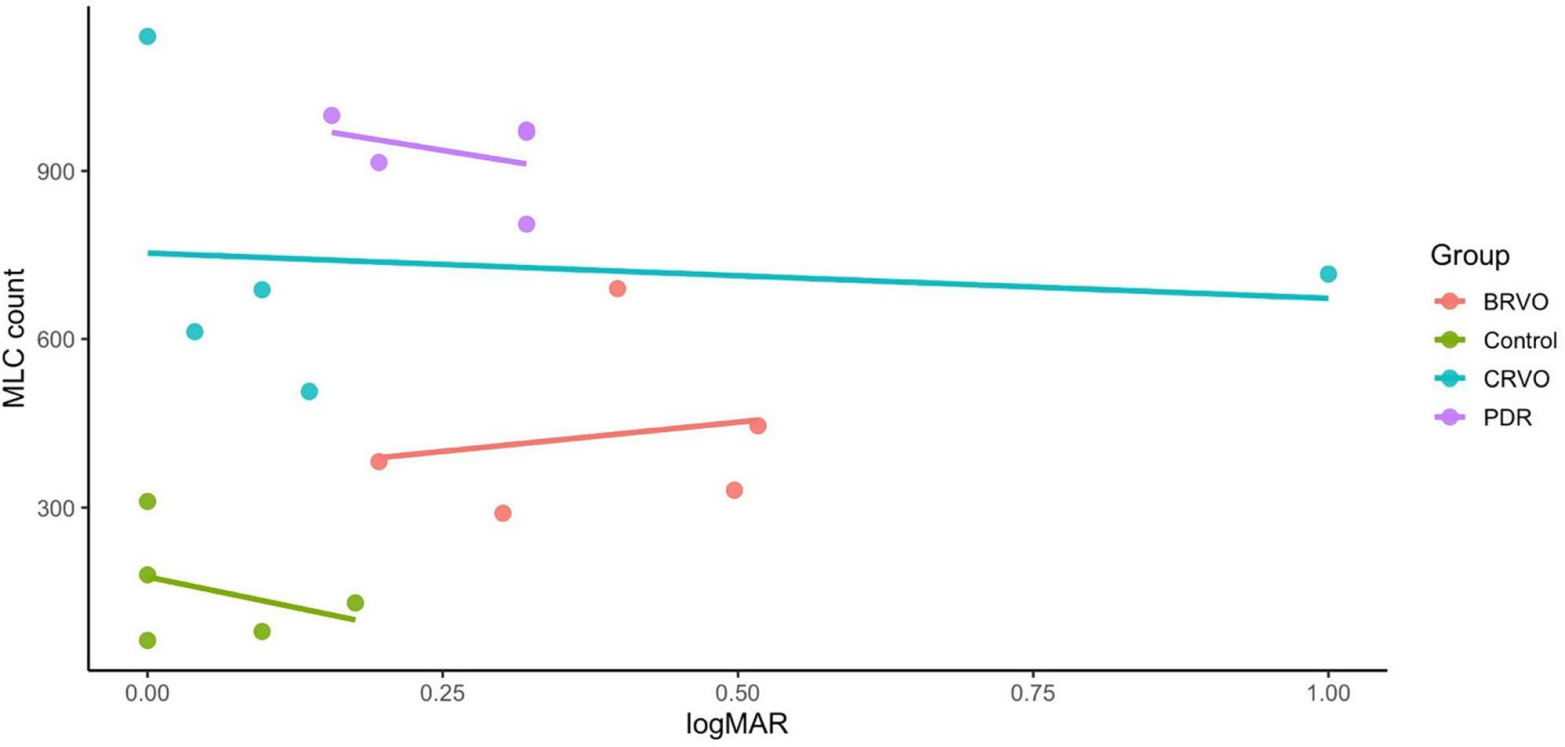
Scatter plot of MLC count versus logMAR visual acuity by group

Lastly, MLC count was evaluated in relation to logMAR visual acuity across groups. Regression analysis did not reveal a consistent positive association between worse visual acuity (higher logMAR) and MLC count (Fig. 6).

## Discussion

In this pilot study we found an increase in the number of MLCs present on OCTA in BRVO, CRVO, PDR, compared to healthy controls. It appears that the number of MLCs increases with the severity of the ischemia, which is more common in PDR. This tendency is seen in the total number of the MLCs as well. The results of this study are in line with the results of Ong et al. and Rangwani et al., who found an increase in the number of MLCs in (proliferative) diabetic retinopathy and retinal vein occlusion compared to healthy individuals [13, 15]. However, these results cannot directly be compared to this study, since the scan area in this study involved the temporal retina and a 6 × 6 mm area, as opposed to the fovea and a 3 × 3 area in other studies [13, 15]. This allows for a broader view and a better understanding of the spatial distribution of MLCs in ischemic diseases of the retina. Moreover, Ong et al. studied the spatial density of MLCs in the macula, with only preliminary findings of a similar pattern of clustering around the blood vessels in the periphery. Here, we confirm their findings in peripheral retina in PDR in addition to CRVO and BRVO patients. In the current study, we were also able to image MLCs in the macula. However, we believe that identifying and quantification of MLCs are inconsistent due to the morphology of the foveal pit. This has been noted in previous studies in the literature, although it is unclear whether MLCs are absent or not successfully imaged around the fovea [13]. Hence, evaluation of the spatial density and quantity of MLCs may be more reliable in the peripheral retina compared to the macula.

Several theories may give an explanation of the increased number of MLCs in vascular diseases. It has been postulated that diabetic retinopathy and/or cardiovascular disease may induce vascular endothelial damage. Consequently, macrophages may migrate due to increased vessel permeability. This may explain a higher number of MLCs near the vessels in patients with retinal vein occlusions or PDR. Conversely, in areas of ischemia, decreased blood flow may lead to a lower number of MLCs with an intravascular origin. It has also been suggested that MLCs may be reliant upon oxidative phosphorylation and thus prefer to avoid the ischemic regions [13]. A similar pattern of increased MLC count in ischemic retinas were observed in the macula as well. However, quantification of MLCs around the foveal pit was deemed as less reliable due to the variability in morphology of the ILM.

With regards to the association between MLC spatial density and retinal perfusion, there was a significant moderate-to-strong negative correlation between MLC count and perfusion density, the significance and effect size of which was most prominent within the deep vascular complex. This result remained largely consistent in a *post hoc* sensitivity analysis using Spearman’s rho, which is less optimal for small sample sizes, where the only different result was that MLC was no longer significantly associated with perfusion density in the superior vascular complex by a small margin. Given this moderate-to-strong negative correlation, it may be hypothesized that MLCs migrate to areas of retinal ischemia which would corresponding to regions of poorer perfusion density.To our knowledge, this finding has not been previously reported in the literature. We hypothesize that areas with a decrease in perfusion density are likely to be the result of extensive vascular disease. This extensive vascular damage may lead to a higher MLC count due to MLC migration and inflammatory processes.

Although this is the first pilot study to look at MLCs in three different diseased states in comparison to healthy subjects, it is not free of limitations. One limitation is the small sample size, with five subjects per group. Another limitation is the lack of detailed clinical information, such as treatment history, systemic comorbidities, and laboratory values, which could act as potential confounders. Moreover, our control group was younger compared to other groups, with a median of 27 as opposed to 71 in BRVO, 72 in CRVO and 52 in the PDR group. Previously, it has been reported that the age group of 70–90 years old is associated with an increased number of MLCs [25]. Although this is a limitation associated with our data, it is noteworthy that the PDR group with a median age of 52 was found to have the highest number of MLCs. Additionally, we did not note an age-associated increase in MLC count within our data, as shown in Fig. 5.

Hence, the disease state is likely to be playing a more major role in MLC’s response. In addition, the image acquisition was limited to the temporal retina, which does not involve a complete MLC count. Inter-rater reliability for image quality ratings between the two graders was fair, with a Cohen’s κ of 0.275 (*p* = 0.226) (Supplementary Table 4). That being said, we are capturing a larger 6 × 6 mm area in this study, which is significantly larger compared to most available studies that consider a 3 × 3 mm field of view. Moreover, it is difficult for us to comment on how the number of MLCs may change over time in association with the chronicity of retinal disease. Hence, MLC count may be increased in patients before a BRVO, CRVO or PDR diagnosis or rather in response to chronic reduced perfusion. However, no significant differences in MLC count have been reported between healthy individuals and patients with diabetes without diabetic retinopathy or with non-proliferative diabetic retinopathy [13]. Finally, with the segmentation of MLCs parts of the retinal vessels may erroneously be included in the MLC count. However, these limitations to segmentation were expected to occur evenly among the four groups.

In conclusion, a higher number of MLCs was observed in BRVO, CRVO and PDR patients compared to healthy control groups. In addition, we observed a negative correlation between perfusion density and MLC count. In combination with results from earlier studies that showed a higher need of intravitreal injections and worse visual outcomes with a higher MLC count, we postulate that the severity of disease may be related to the number of MLCs that are present. Monitoring MLCs may be a potential biomarker for disease progression, while modulating their response presents a novel avenue in management of ischemic retinal disease.

## Supplementary Information

Below is the link to the electronic supplementary material.

Supplementary Material 1

## Data Availability

No datasets were generated or analysed during the current study.
