# Supplementary material for: Evaluating the quantity and spatial density of macrophage-like cells in patients with retinal vascular disease and healthy subjects via non-invasive retinal imaging
Source: Int J Retina Vitreous. 2025 Oct 9;11:104. doi: 10.1186/s40942-025-00729-7 (PMC12512729; doi:10.1186/s40942-025-00729-7)
Supplement: Supplementary file 1 — Supplementary Material 1 [file 40942_2025_729_MOESM1_ESM.docx]

**Supplemental Table 1.** Individual patient-level demographics and MLC count across groups (Control, PDR, CRVO, BRVO).

**Supplemental Table 2.** Difference in cell counts between the diseased and the control group. Using the bootstrap method

| **Group** | **Median Difference vs Control** | **Percentile CI (Low–High)** | **BCa CI (Low–High)** |
| --- | --- | --- | --- |
| **BRVO** | **252** | **71 – 560** | **–21 – 303** |
| **CRVO** | **558** | **327 – 1010** | **196 – 558** |
| **PDR** | **839** | **625 – 910** | **494 – 789** |

**Supplemental Table 3.** Vascular plexus and flow associations

| Vascular Plexus | **τ (Kendall’s tau)** | **Uncorrected p-value** | **Bonferroni-corrected p-value** | **Significance After Correction**  **(**Bonferroni) |
| --- | --- | --- | --- | --- |
| Superior | - 0.49 | 0.04 | 0.12 | Not Significant |
| Deep | - 0.64 | <0.01 | 0.027 | Significant |
| Combined (Superior Deep) | - 0.60 | 0.01 | 0.030 | Significant |

**Supplemental Table 4**. Interrater reliability for temporal 6x6 mm scans

| T1 | T2 |
| --- | --- |
| Good | Good |
| Good | Blurry |
| Good | Blurry |
| Good | Good |
| Good | Good |
| Good | Good |
| Good | Good |
| Blurry | Blurry |
| Blurry | Good |
| Good | Good |
| Good | Good |
| Good | Good |
| Blurry | Good |
| Blurry | Blurry |
| Good | Good |
| Good | Good |
| Blurry | Good |
| Good | Good |
| Good | Good |


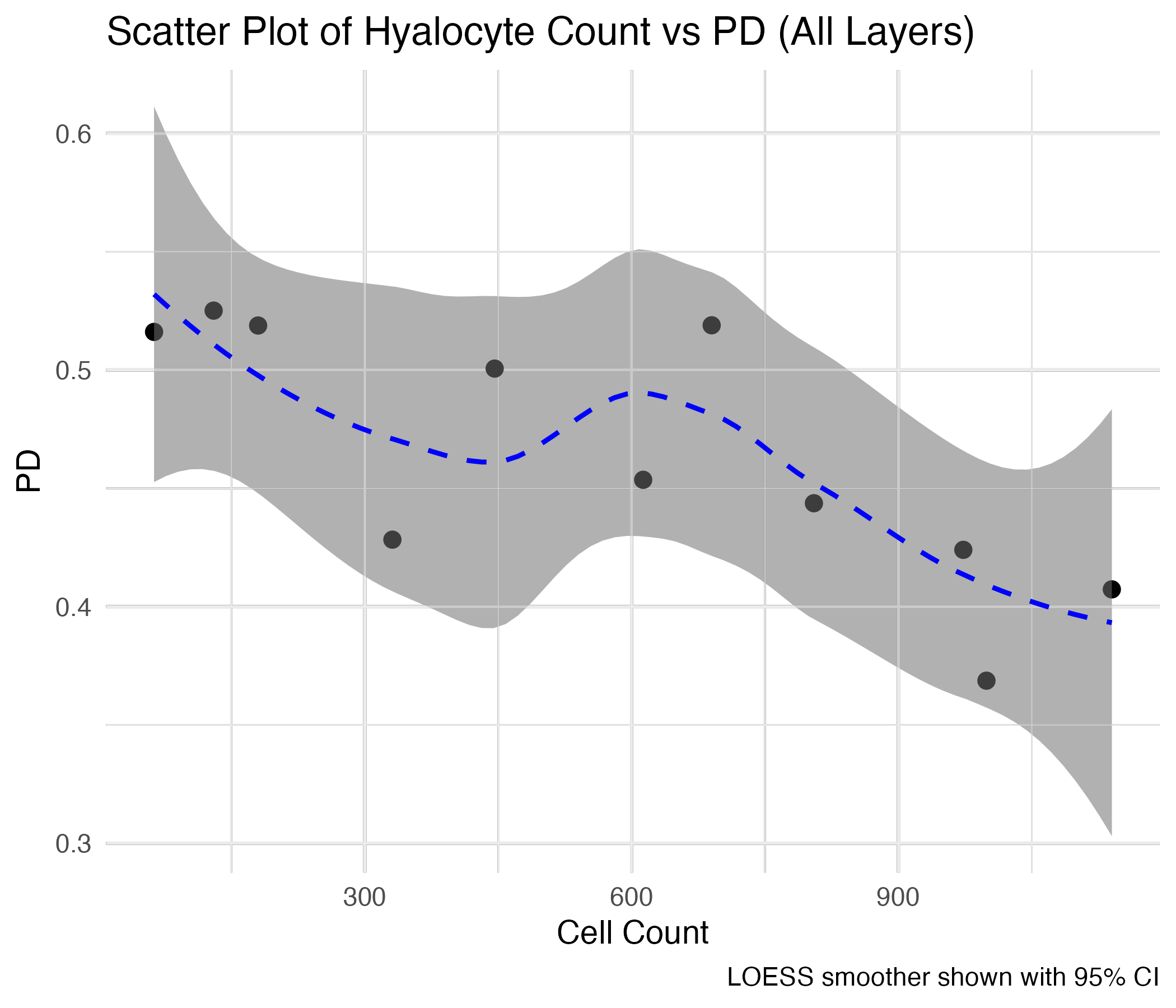


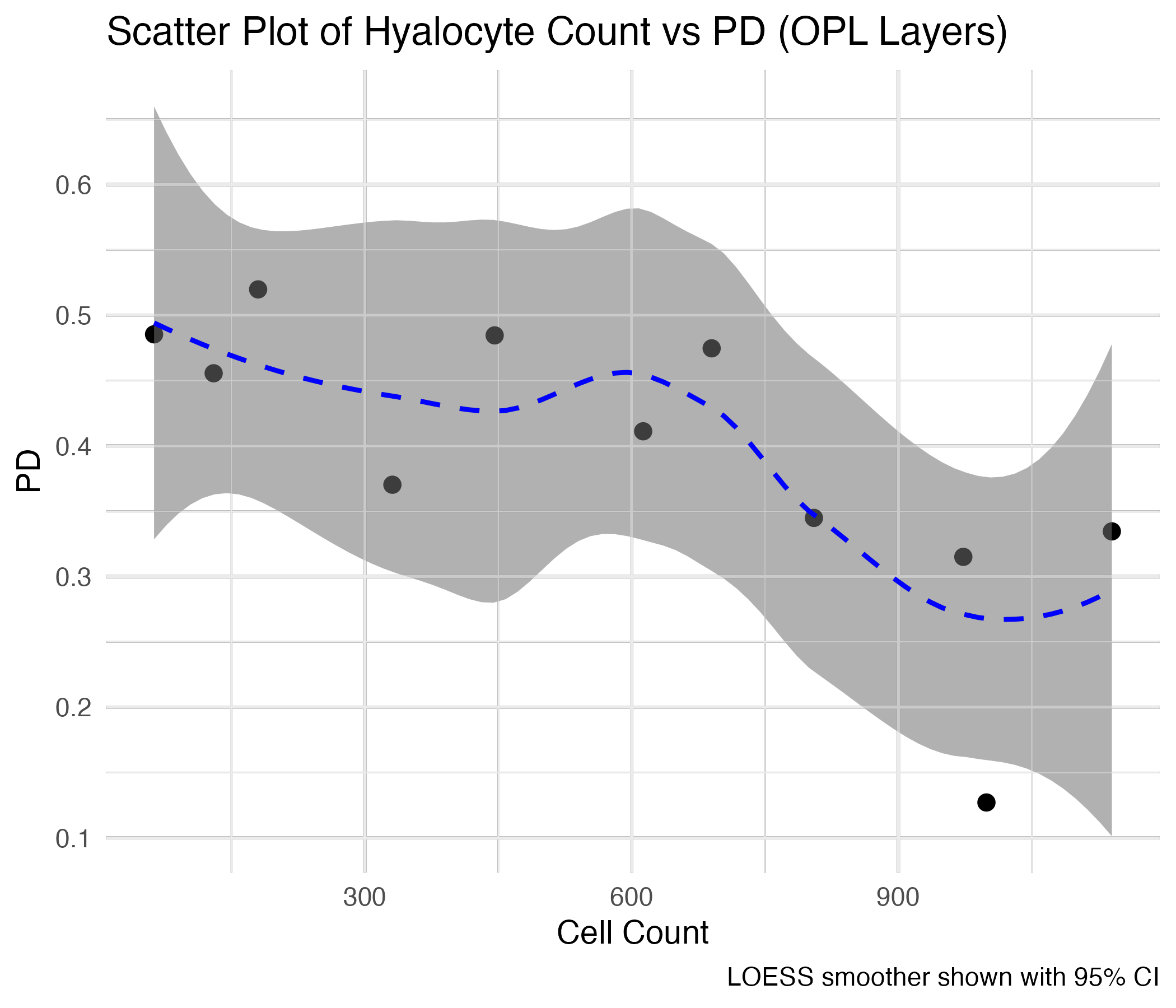


**C.**


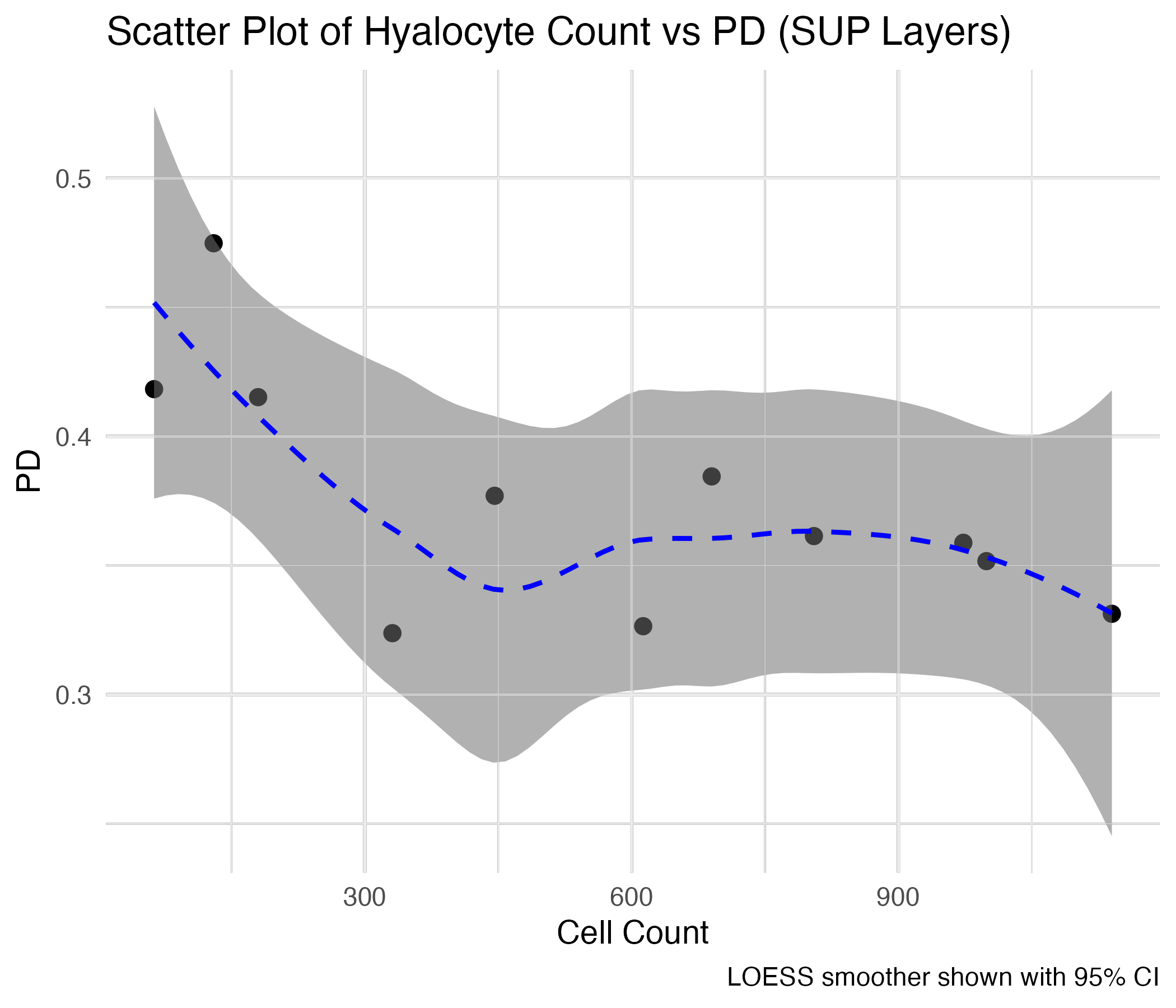


**Supplemental Figure 1.** Scatter plot showing the relationship between MLC count and perfusion density across all layers (A), OPL layers (B), and SUP layers (C).
